# Evaluation of phase-adjusted interventions for COVID-19 using an improved SEIR model

**DOI:** 10.1017/S0950268823001796

**Published:** 2023-11-13

**Authors:** Honglin Jiang, Zhouhong Gu, Haitong Liu, Junhui Huang, Zhengzhong Wang, Ying Xiong, Yixin Tong, Jiangfan Yin, Feng Jiang, Yue Chen, Qingwu Jiang, Yibiao Zhou

**Affiliations:** 1 Fudan University School of Public Health, Shanghai, China; 2 Key Laboratory of Public Health Safety, Fudan University, Ministry of Education, Shanghai, China; 3 Fudan University Center for Tropical Disease Research, Shanghai, China; 4 Fudan University School of Computer Science and Technology, Shanghai, China; 5 School of Public Health, Hebei Medical University, Shijiazhuang, China; 6 School of Epidemiology and Public Health, Faculty of Medicine, University of Ottawa, Ottawa, ON, Canada

**Keywords:** COVID-19, incubation period, non-pharmacological interventions, quarantine, SEIR model

## Abstract

A local COVID-19 outbreak with two community clusters occurred in a large industrial city, Shaoxing, China, in December 2021 after serial interventions were imposed. We aimed to understand the reason by analysing the characteristics of the outbreak and evaluating the effects of phase-adjusted interventions. Publicly available data from 7 December 2021 to 25 January 2022 were collected to analyse the epidemiological characteristics of this outbreak. The incubation period was estimated using Hamiltonian Monte Carlo method. A well-fitted extended susceptible-exposed-infectious-recovered model was used to simulate the impact of different interventions under various combination of scenarios. There were 387 SARS-CoV-2-infected cases identified, and 8.3% of them were initially diagnosed as asymptomatic cases. The estimated incubation period was 5.4 (95% CI 5.2–5.7) days for all patients. Strengthened measures of comprehensive quarantine based on tracing led to less infections and a shorter duration of epidemic. With a same period of incubation, comprehensive quarantine was more effective in containing the transmission than other interventions. Our findings reveal an important role of tracing and comprehensive quarantine in blocking community spread when a cluster occurred. Regions with tense resources can adopt home quarantine as a relatively affordable and low-impact intervention measure compared with centralized quarantine.

## Introduction

Coronavirus disease 2019 (COVID-19), caused by severe acute respiratory syndrome coronavirus 2 (SARS-CoV-2), has swept the globe since it broke out in Wuhan, China, in December 2019. As of 28 November 2022, there were over 637 million confirmed cases and over 6 million deaths in 228 countries and territories [1], including 315,248 confirmed cases reported in mainland China [2].

After successfully controlling the first wave of the pandemic in Wuhan in late March 2020, China has implemented multilayer non-pharmacological interventions (NPIs) to contain sporadic and localized outbreaks and maintain low infection rates in the general population [3]. The experience in the fight against COVID-19 highlights the critical roles of contact tracing, isolation and mass testing [4], which are deployed as a comprehensive set of NPIs in pursuit of the dynamic zero-COVID strategy [5]. However, these strict NPIs could come at high economic and social costs, albeit with a rapid fall in the peak epidemic size [6]. Moreover, vaccine-induced population immunity may be insufficient to prevent COVID-19 outbreaks in the context in which excess mutations of SARS-CoV-2 have produced sets of highly transmissible omicron variants [7, 8]. Thus, regions and countries should impose tailored management adapted to epidemic dynamics at different levels of stringency in close temporal sequence.

Susceptible-exposed-infectious-recovered (SEIR) is a widely used epidemiological framework to characterize the epidemic dynamics of an infectious disease. As NPIs change the course of SARS-CoV-2 transmission [9], many modified SEIR models [10–14] have been proposed to evaluate the effects of various measures and project the future trajectory. However, due to various factors across different regions such as economic, government policy and vaccine coverage, no compartmental model appears to be the best for all scenarios. In addition, continuously implemented NPIs of various levels could impact the epidemic trend over time. Despite several studies have preliminarily modelled phase-adjusted projection for COVID-19 outbreaks [15–17], little has dynamically evaluated these measures within the SEIR framework. A quantitative comparison of the effectiveness of existing integrated NPIs for tracing, quarantining and screening during an ongoing outbreak is lacking, and the contribution of different quarantine strategies in community setting was not distinguished in previous studies.

Shaoxing, a city in northern Zhejiang Province, China, emerged a COVID-19 cluster related to a funeral in early December 2021 [18]. Unlike previous localized outbreaks, a second cluster occurred after serial interventions were imposed for a period of time. We established an improved susceptible-exposed-infectious-asymptomatic-recovered (SEIAR) model to simulate the COVID-19 transmission dynamics in Shaoxing using publicly reported data and estimated the period of incubation. Effectiveness of case/contact tracking and quarantine strategies at different stages of intervention were considered into model fitting. This observed, phase-adjusted model was then used to make inference about the magnitude of COVID-19 infections under different combinations of existing measures.

## Methods

### Data source

Data concerning all SARS-CoV-2-infected cases in Shaoxing were publicly available on the official website of the Health Commission of Shaoxing (http://sxws.sx.gov.cn/) and WeChat public account of Shaoxing government (http://m.shaoxing.com.cn/z/2881390/). Data from 7 December 2021 to 25 January 2022 on daily numbers of new confirmed cases, asymptomatic carriers, confirmed cases transferred from asymptomatic infection and recovery/death were obtained. Information including characteristics of infected persons (gender, age, district of residence), possible exposure period and dates of initial diagnosis, symptom onset and isolation/quarantine were also collected.

### Case definition

All infected cases were diagnosed according to the Diagnosis and Treatment Protocol for Novel Coronavirus Pneumonia (Trial Version 8, Revised) [19] released by the National Health Commission of China & State Administration of Traditional Chinese Medicine on 14 April 2021. Individuals who had clinical manifestations (e.g. fever, respiratory symptoms, CT imaging characteristics) and were with a positive nucleic acid amplification test (NAAT) result were immediately diagnosed as confirmed cases. Those tested positive for nucleic acid but had no COVID-19 symptoms at first were diagnosed as asymptomatic infected. Some of them might later develop symptoms and become confirmed (symptomatic) cases.

In this study, we classified daily new confirmed cases into two categories: immediately confirmed cases and later confirmed cases. Immediately confirmed cases refer to individuals who manifested clinical symptoms or CT results when tested positive for nucleic acid. Later confirmed cases are those initially diagnosed as asymptomatic carriers but later developed symptoms. The sum of immediately confirmed and later confirmed cases per day was defined as the number of daily new infections.

### Interventions in Shaoxing

For the goal of precise control, communities/villages were classified as sealed-off area, control area and prevention area, according to the risk level of transmission. Different strategies were adopted in each area to prevent COVID-19 spread. For sealed-off areas, home isolation measure was completely implemented with door-to-door service provided. In control areas, residents were not allowed to leave the area, and gathering was strictly prohibited. Social interventions were strengthened and gathering activities were restricted in prevention areas.

To contain the ongoing epidemic, a series of intensified prevention and control measures was successively implemented in Shaoxing, including school closure, transportation suspension, lockdown of epidemic district, building of centralized quarantine houses and city-wide mass NAAT (Supplementary Table S1). As of 31 December 2021, the outbreak has basically ended by achieving ‘dynamic zero-case’, and the protective measures and restrictions were eased back to the level in the normalization stage. Here, to better simulate the transmission dynamics and assess the effects of control measures, we divided the implementation of interventions from 7 to 31 December into three stages based on the dates of key events.

#### The first stage (7–10 December)

The interventions were mainly implemented in Shangyu District, the epicentre of this outbreak where the first case was reported on 7 December. On 9 December, Shaoxing upgraded its COVID-19 emergency response to the highest level, with one new case reported in Yuecheng District. On 10 December, Shangyu District launched its first round of mass NAAT.

#### The second stage (11–15 December)

From 11 December, a city-wide NAAT was activated in Shaoxing. Apart from Shangyu, other districts started to conduct their first round of mass NAAT. Meanwhile, Shangyu District was locked down and all entrances and exits were closed. As of 15 December, three rounds of district-wide NAAT had been performed in Shangyu.

#### The third stage (16–31 December)

On 16 December, the first batch of 5,025 sets of centralized isolation houses in Shaoxing was built, followed by the largest temporary quarantine site with 600 beds in Shangyu put into use on 17 December. During this stage, Shangyu conducted another four consecutive mass NAATs. On 31 December, the lockdown in Shangyu District was lifted. The whole city regulated its risk level to the lowest. Since then, all the control measures such as home quarantine for community residents and closure of public places were lifted.

### Statistical analysis

Epidemic curves for the dates of confirmed diagnosis and the timeline of key interventions in Shaoxing were drawn. Infected cases with known dates of symptom onset and available exposure information were selected for the estimation of incubation period. The characteristics of immediately confirmed cases and later confirmed cases among these people were compared using *t*-test or Chi-squared/Fisher’s exact test. Two-tailed *P* values <0.05 were considered statistically significant.

Incubation period is the number of days between one’s exposure to SARS-CoV-2 and the onset of symptoms. The possible exposure period was calculated as the interval between the earliest possible exposure date and the latest possible exposure date. Three parametric distributions (Weibull, Gamma and Log-normal) were fitted for the period of incubation using Hamiltonian Monte Carlo method for Bayesian inference [20]. Mean, SD, 95% CI for each incubation period were estimated. The best-fit model was evaluated using the Leave-One-Out Information Criterion (LooIC). Statistical analyses were conducted with R 4.1.2.

### COVID-19 transmission modelling

**Figure 1.**
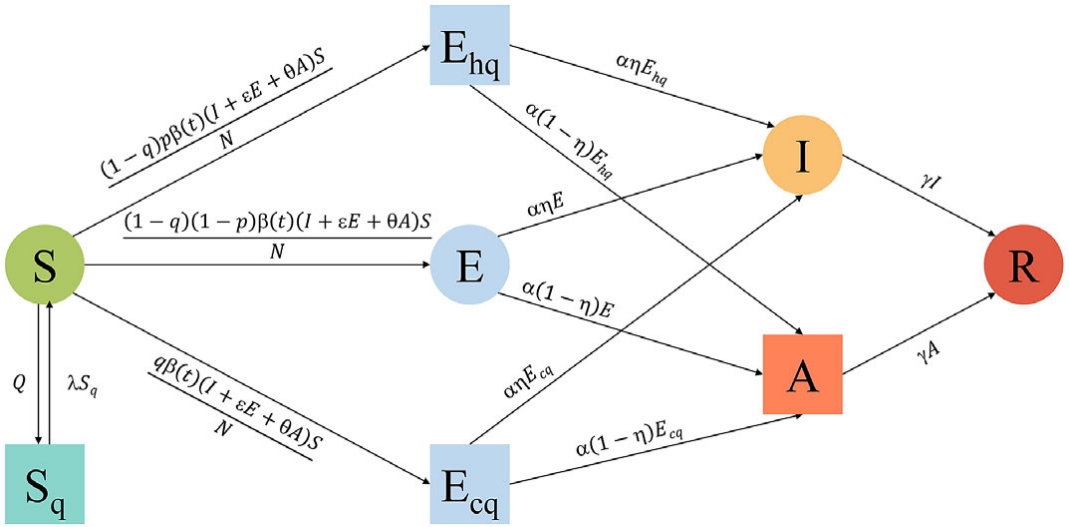
The structure of SEIAR model and the relationships between different compartments. SEIAR, susceptible-exposed-infectious-asymptomatic-recovered. Black arrows show movements among compartments. These movements are illustrated by formulas with assumed parameters that inform different interventions. In this flowchart, we separated the symptomatic and asymptomatic infections and the exposed persons based on whether they were being traced, centralized quarantined or home-quarantined.

A classic SEIR compartmental framework with Bayesian underpinning was applied to model the COVID-19 transmission dynamics in Shaoxing. Considering the infectivity of asymptomatic carriers and the effects of different interventions on the outbreak, the SEIR model was extended to the SEIAR model (Figure 1).

Four additional compartments were introduced into the modified model: asymptomatic (*A*), comprehensive quarantined susceptible (*S_q_*), centralized quarantine exposed (*E_cq_*) and home quarantine exposed (*E_hq_*). The initial values of compartments were set according to the released number of cases. The SEIAR model was fitted on dynamically changing data, including daily numbers of immediately confirmed cases, asymptomatic carriers and cases removed from the model. The limited-memory Broyden-Fletcher-Goldfarb-Shanno (L-BFGS) algorithm [21] was used to find the best estimation of unknown parameters by fitting them to find the zero solution of the equations. Epidemic curve fitting was then performed based on the actual number of cumulative infections. Equations for the change of each compartment size were built as follows:

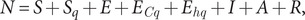




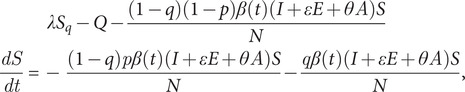




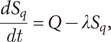




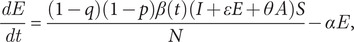




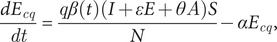




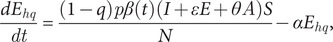




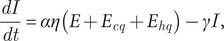




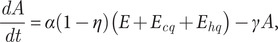




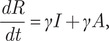

where *Q* represents the number of quarantined susceptible people per day, *λ* represents the rate of release from quarantine (i.e. 1/isolation period), *q* represents the probability of an exposed person being traced and centralized quarantined, *p* represents the probability of exposed people being home-quarantined, *β*(*t*) represents the number of newly infected susceptible persons over time (i.e. transmission velocity), *α* represents the transition rate of latency to infectiousness (i.e. 1/incubation period), *ε* and *θ* represent the relative transmissibility of exposed people and asymptomatic carriers, respectively, and *η* represents the proportion of symptomatic people in positive infections. Because of no death, *γ* represents the rate of recovery (i.e. 1/recovery time).

We also calculated the reproduction number (*R*) for each estimated *β*(*t*) based on Zhou et al. deduction [22]:

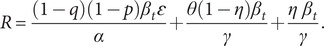



The fitted SEIAR model was used to simulate and assess the impacts of different interventions. Among the parameters (*Q*, *λ*, *q*, *p*), *Q* and 1/*λ* denoted the scale and time of comprehensive quarantine (including centralized quarantine and home quarantine), and *q* was determined by the effectiveness of epidemiological investigation and mass NAAT for case/contact tracing. As residents in sealed-off areas were required to be isolated at home, *p* also reflected the strictness of control measures at the community level. Model fitting and scenario simulation under different combinations of interventions were performed in Python 3.9.0.

## Results

### Characteristics of patients

A total of 387 SARS-CoV-2-infected cases were identified from 7 to 27 December 2021 for the Shaoxing outbreak (384 cases in Shangyu District and 3 in Yuecheng District) (Supplementary Figure S1). As of 25 January 2022, 350 patients recovered and were discharged from hospital. Of the 387 cases, 355 (91.7%) were immediately confirmed and 32 (8.3%) at their pre-symptomatic stage (later confirmed cases).

A data subset containing 204 patients with demographic and exposure information was created. These patients were identified before 16 December and aged 48.9 years on average. There was a greater proportion of immediately confirmed cases for female patients, but a greater proportion of later confirmed cases for male patients (*P* < 0.001). No significant difference in age was found between immediately confirmed cases and later confirmed cases. There was a high rate of quarantine before diagnosis for both groups. However, most of the later confirmed cases were found during a centralized quarantine, while a larger percentage of immediately confirmed cases were found during a home quarantine (*P* < 0.001) (Table 1).

**Table 1.** Characteristics of 204 COVID-19 cases in Shaoxing epidemic

	Group	All cases (*n* = 204)	Immediately confirmed cases (*n* = 175)	Later confirmed cases^a^ (*n* = 29)	*P*-value
Gender	Male	94 (46.1)	78 (44.6)	16 (55.2)	<0.001
	Female	101 (49.5)	94 (53.7)	7 (24.1)	
	Unknown	9 (4.4)	3 (1.7)	6 (20.7)	
Age (years)		48.9 ± 17.4	49.1 ± 17.6	46.8 ± 16.2	0.327
Case finding	Centralized quarantine	107 (52.5)	81 (46.3)	26 (89.7)	<0.001
	Home quarantine	80 (39.2)	79 (45.1)	1 (3.4)	
	Active NAAT^b^	17 (8.3)	15 (8.6)	2 (6.9)	
Being isolated before diagnosis	Yes	187 (91.7)	160 (91.4)	27 (93.1)	0.762
	No	17 (8.3)	15 (8.6)	2 (6.9)	

Age is described as mean ± SD, and others are shown as numbers and percentages. Comparisons were conducted between immediately confirmed cases and later confirmed cases.

NAAT, nucleic acid amplification test.

a Later confirmed cases also refer to initially diagnosed asymptomatic cases.

b Active NAAT includes mass NAAT, routine NAAT and NAAT for key population.

### Estimation of incubation period

Three distribution models showed the same average periods of incubation for all cases and immediately confirmed cases, which were 5.4 (95% CI 5.2–5.7) days and 5.2 (95% CI 5.0–5.4) days, respectively. According to LooIC values, the Log-normal distribution presented the best fits to the data for all cases (LooIC = 761.5) and immediately confirmed cases (LooIC = 608.1). Weibull distribution fit the data best for later confirmed cases (LooIC = 126.1), estimating a significant higher average period of incubation (7.2, 95% CI 6.4–7.9 days) than immediately confirmed cases (*P* < 0.001) (Table 2 and Supplementary Figure S2).

**Table 2.** Mean and SD of the estimated incubation period for COVID-19 cases in Shaoxing epidemic

	Distribution	Estimated incubation period (d)		LooIC	Parameters
		Mean (95% CI)	SD (95% CI)		
All infected cases (*n* = 204)	Weibull	5.4 (5.2, 5.7)	1.6 (1.5, 1.8)	790.5	*α* = 3.75, *σ* = 6.01
	Gamma	5.4 (5.2, 5.7)	1.5 (1.3, 1.6)	766.6	*α* = 14.05, *β* = 2.59
	Log-normal	5.4 (5.2, 5.7)	1.5 (1.3, 1.7)	761.5	*μ* = 1.66, *σ* = 0.27
Immediately confirmed cases (*n* = 175)	Weibull	5.2 (5.0, 5.4)	1.4 (1.3, 1.6)	631.0	*α* = 4.17, *σ* = 5.72
	Gamma	5.2 (5.0, 5.4)	1.3 (1.1, 1.4)	612.3	*α* = 17.15, *β* = 3.30
	Log-normal	5.2 (5.0, 5.4)	1.3 (1.1, 1.5)	608.1	*μ* = 1.62, *σ* = 0.25
Later confirmed cases (*n* = 29)	Weibull	7.2 (6.4, 7.9)	1.9 (1.5, 2.5)	126.1	*α* = 4.39, *σ* = 7.88
	Gamma	7.0 (6.3, 7.9)	1.8 (1.4, 2.5)	129.7	*α* = 15.03, *β* = 2.13
	Log-normal	7.1 (6.3, 8.1)	2.2 (1.6, 3.3)	131.2	*μ* = 1.91, *σ* = 0.31

LooIC, Leave-One-Out Information Criterion.

Three distributions (Weibull, Gamma and Log-normal) were used to estimate the incubation periods for all cases, immediately confirmed cases and later confirmed cases, respectively. 95% CI was also calculated for both mean and SD of each estimate. Data were considered to fit best with the smallest value of LooIC index. For all infected cases and immediately confirmed cases, the Log-normal distribution provides the best fit to data, while the Weibull distribution shows best fit data for later confirmed cases.

Our results also show a larger variation in incubation period for later confirmed cases than all cases and immediately confirmed cases (Supplementary Table S2). The estimated median of incubation period for later confirmed cases using Weibull distribution was 7.2 days, ranging from 3.4 (0.025th) to 10.6 days (0.975th). While the 0.025th to 0.975th percentiles of incubation periods estimated by Log-normal distribution were 3.1 to 8.9 days for all cases and 3.1 to 8.2 days for immediately confirmed cases. Although the variation of incubation period existed, all patients developed symptoms within 14 days of the earliest possible exposure.

### SEIAR modelling

**Figure 2.**
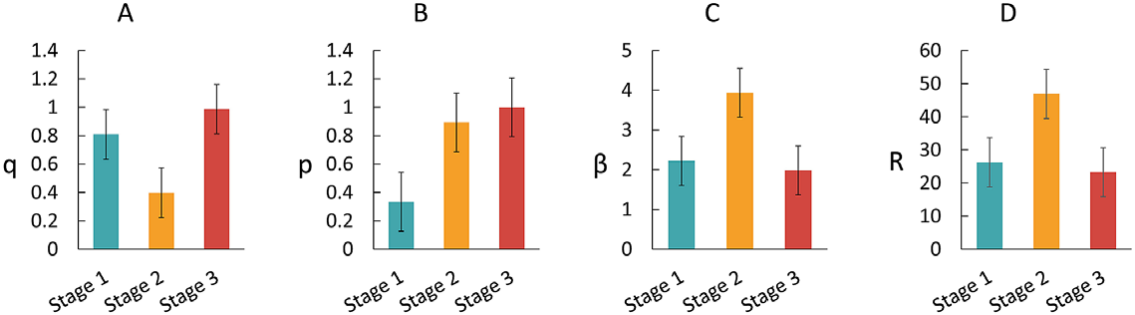
Changes in effectiveness of control measures and transmission velocity in three stages of intervention. (a) *q* is the probability of an exposed person being traced and under centralized quarantine; (b) *p* is the probability of an exposed person being home-quarantined; (c) *β* is transmission velocity; and (d) *R* is reproduction number.

**Figure 3.**
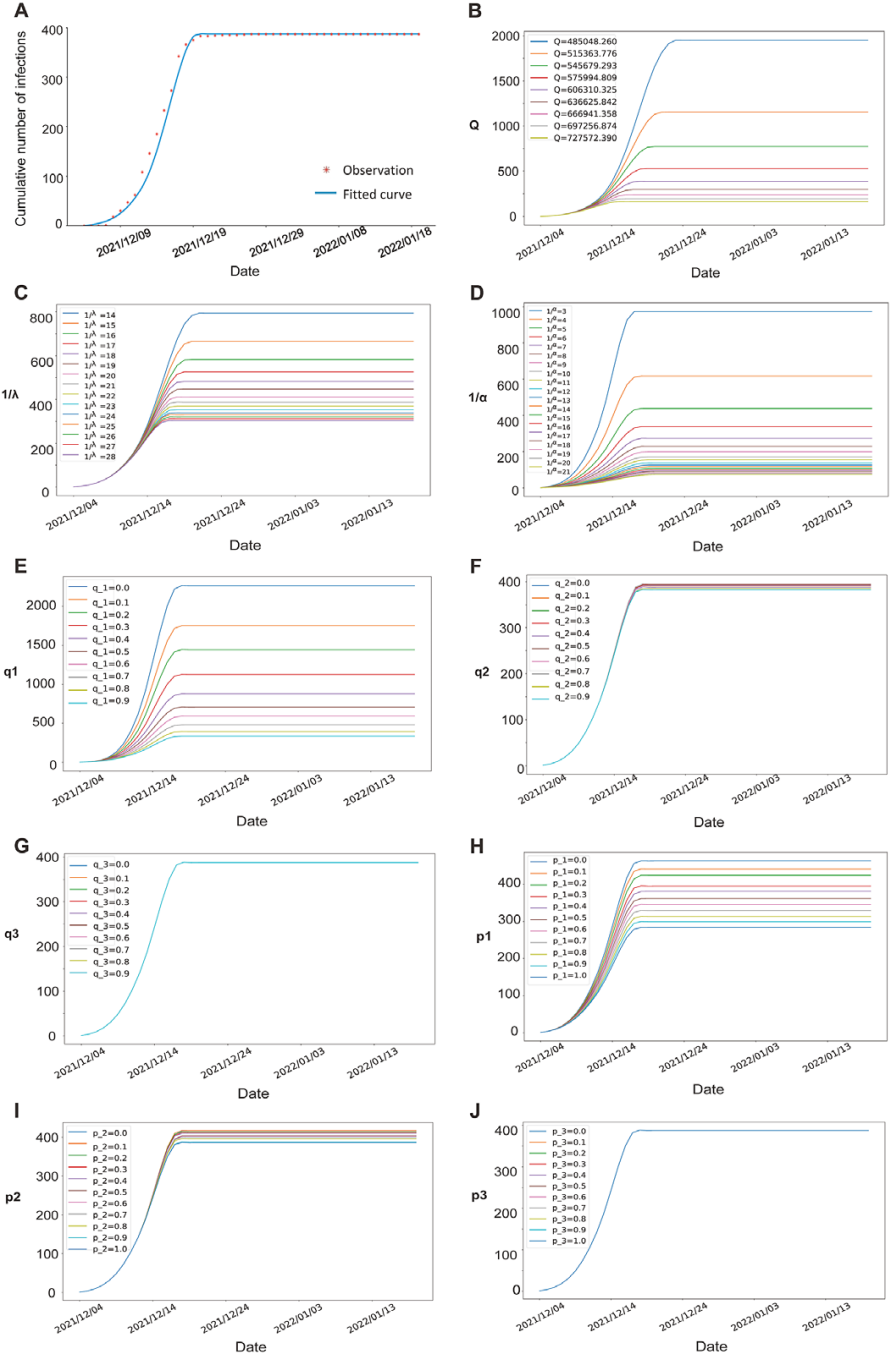
(a–j) SEIAR model fitting and simulation. There have been no newly infected cases since 27 December 2021. We fitted the model utilizing the data including the daily number of infections and the number of people recovered, from the date of first case reported to 18 January 2022. (a) SEIAR model fitting based on cumulative number of infected cases. Red asterisk, observation; blue line, fitted curve. Plots (b–j) show the effects of different interventions on the cumulative number of infections. (b) Comprehensive quarantine (*Q*). (c) Isolation period (1/*λ*). (d) Incubation period (1/*α*). (e) Tracing and centralized quarantine in the first stage (*q*1). (f) Tracing and centralized quarantine in the second stage (*q*2). (g) Tracing and centralized quarantine in the third stage (*q*3). (h) Home quarantine in the first stage (*p*1). (i) Home quarantine in the second stage (*p*2). (j) Home quarantine in the third stage (*p*3).

Theoretical transmission dynamics of COVID‐19 based on the SEIAR model were simulated. Control measures were adjusted at different stages in response to the outbreak, which could influence the susceptibility of the general population. Given the varying intensity of different measures, we calculated the values of *q* and *p* in the three stages of intervention to obtain optimal *β* and the corresponding *R* (Supplementary Table S3). The spread of COVID-19 was fastest during the second stage (*β* = 3.9356, *R* = 46.9048) when *q* was at the lowest level. With both *q* and *p* being highest in the third stage, the transmission speed decreased to the lowest (*β* = 1.9845, *R* = 23.2556) (Figure 2). Supplementary Table S4 presents other estimated parameters. The SEIAR model shows a good fit to the actual data (Figure 3a).

### Scenario simulation

The impacts of different interventions (*Q*, *λ*, *q*, *p*) on the cumulative number of infections was simulated by assuming one varied parameter with others staying still during the three stages. To assess the effectiveness of comprehensive quarantine measures, *Q* was set ranging from 0.8 to 1.2 times of its initial value, and 1/*λ* ranged from 14 to 28 days. Stricter quarantine measures or longer periods of isolation could result in slower growth of cases, smaller number of total infections and shorter duration of epidemic (Figure 3b,c).

To assess the effects of adjustments to interventions during the outbreak, different levels of *q* and *p* (both ranged from 0 to 1) were simulated in each stage (Figure 3e–j). During the first and second stages, if more exposed/infected people had been found and under centralized quarantine (higher *q*), or isolated at home (higher *p*) before being infectious, less susceptible persons would be infected (Figure 3e–h). The impact of changes in one control measure was also determined by the other. With lower *p* in the first stage (compared with corresponding *q*) or lower *q* in the second stage, the variation in the effect of varied *q* was larger than that of *p* in stage 1 (Figure 3e,h) but smaller in stage 2 (Figure 3f,i). Notably, the effectiveness of either *q* or *p* measures had little effect on the duration of the epidemic.

We also simulated different lengths of incubation periods (1/*α*) with a range of 3–21 days. A longer period of incubation reduced the cumulative number of cases, but prolonged the duration of the epidemic (Figure 3d).

**Figure 4.**
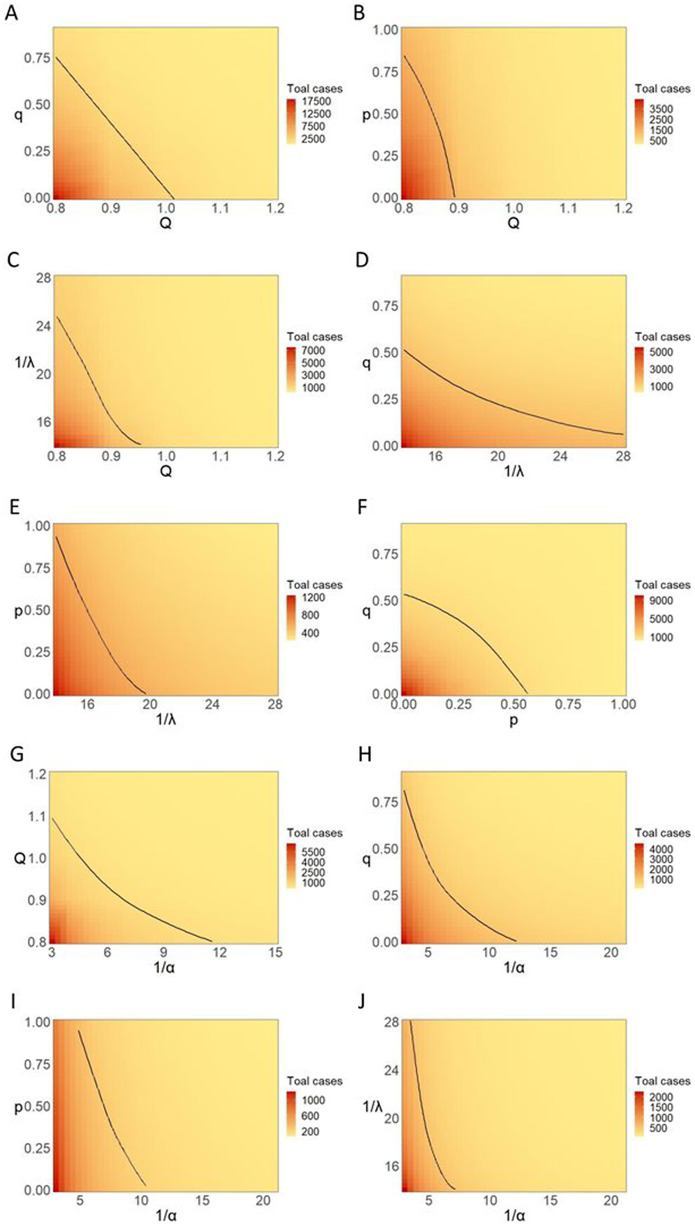
(a–j) Effects of different combinations of interventions on the cumulative number of infections. *Q*, comprehensive quarantine; 1/*λ*, isolation period; 1/*α*, incubation period; *q*, tracing and centralized quarantine; *p*, home quarantine.

The results of different combinations of interventions show that enhancing the measure of comprehensive quarantine was more powerful in controlling the transmission than other interventions; the smaller the number of general exposed population being isolated, the much longer period of isolation or the earlier tracing and management of infected persons needed (Figure 4a–c). If the period of isolation declined, tracing and centralized quarantine of infected cases were more important than quarantine at home. In other words, epidemiological investigation and mass NAAT should be more efficient (Figure 4d–f).

Incubation period could also influence the implementation of containment measures (Figure 4g–j). More rigorous tracing and quarantine strategies were required when the latent time was shorter. With the same period of incubation, comprehensive quarantine measures had the largest impacts on the number of infections, followed by the effectiveness of tracing and centralized quarantine for exposed people (Figure 4g,h). The effect of isolation period on epidemic spreading was limited (Figure 4j).

## Discussion

We explored a new SEIAR model to better understand the influences of NPIs on COVID-19 outbreak in Shaoxing. First, an additional compartment *A* (i.e. asymptomatic when screening) was introduced into the traditional SEIR model, consistent with previous studies [23–25]. Asymptomatic individuals accounted for over half of COVID-19 transmissions, including 35% from pre-symptomatic persons [26]. Delayed detection can lead to a large increase in disease prevalence [27], while control interventions may influence the proportion of pre-symptomatic infections. We estimated the infectivity of initial asymptomatic carriers at 58% of symptomatic patients, indicating that introducing *A* was important to derive a general model for better understanding of COVID-19 transmission.

Other three compartments relevant to NPIs were also incorporated: *S_q_*, *E_cq_* and *E_hq_.* A proportion of exposed individuals found by contact tracing would be quarantined (either centralized or home quarantine). They could be moved to *S_q_* or *E_cq_*/*E_hq_* based on whether they were effectively infected. Those exposed to the virus but missed by contact tracing would be moved to *E.* The strictness of quarantine was generally made at the regional level by local government officials. Shaoxing was more likely to impose home quarantine rather than centralized quarantine for secondary close contacts. However, infected persons among them might spread the virus within their households despite limited transmission coverage. Several modelling studies [13, 14, 28] assessing the impacts of quarantine and other NPIs did not differentiate centralized quarantine from home quarantine. We set up two compartments (*E_cq_* and *E_hq_*) to separate exposed people under centralized and home quarantines. A relatively high *q* but much lower *p* values during the first stage of intervention in Shaoxing indicated that a considerable proportion of infected people had not been traced and quarantined before mass testing. It might increase the possibility of community spread and be a reason for the second cluster in a supermarket [29]. During the second stage, more infected persons were found among those under home quarantine (high *p*) than among those under centralized quarantine (low *q*). It showed that home isolation could be complementary to centralized quarantine to mitigate community spread.

Our findings also demonstrate that mass NAAT substantially improved the effectiveness of case tracing. The number of infections reached a new peak at the beginning of the third stage and then decreased with no resurgence. The high value of *q* in the third stage suggests that multiple rounds of mass NAAT had facilitated early identification and isolation of infected cases.

Comprehensive quarantine measures refer to different types of quarantine, including individual quarantine, centralized quarantine and home isolation for exposed population (e.g. close contacts, general contacts). SEIAR-driven simulations imply that tracing and quarantine of infected individuals mainly influenced the magnitude of an outbreak, while comprehensive quarantine measures could affect the duration of the epidemic. It was demonstrated by another study that highly effective contact tracing and case isolation can control a new COVID-19 outbreak within 3 months, and the probability of control decreases with long delays from symptom onset to isolation [30]. Comprehensive quarantine of exposed people had a more significant effect on the outbreak than a single quarantine measure for infected people. Derivative scenarios suggest that when an outbreak originates from a cluster, the intensity of quarantine needs to be increased to block SARS-CoV-2 transmission. A systematic review has suggested that adhering only to screening and isolation with lower coverage is not effective enough to reduce the epidemic; quarantine should be implemented early and must cover a larger community [31]. To reduce economic and other social costs, stringent home isolation could be a good choice in regions with tense resources, combined with simultaneous construction of quarantine centres, contact tracing and mass testing.

We found a shorter incubation period for COVID-19. A higher transmission speed asks for faster tracing and quarantine of contagious people. The incubation period also serves as a determinant of quarantine duration for exposed persons [32]. Currently, most cities in China, including Shaoxing, have extended quarantine time to ‘14 plus 7’ days (compulsory 14-day centralized quarantine and 7-day home quarantine or additional centralized quarantine), as several studies have reported an observed incubation period longer than 14 days [33]. We estimated an average incubation period of 5.4 days in this outbreak. The mean incubation period of later confirmed cases was longer than that of immediately confirmed cases, but 97.5 quartiles of patients had a less than 10-day latency. Thus, it may be feasible to shorten the period of centralized quarantine for those close contacts to release the isolation burden on both individuals and the public health system.

In this study, we used the classical SEIR model with an additional ‘asymptomatic’ component, which is in line with the transmission mechanism of COVID-19. To precisely simulate the epidemic trajectory, *S* and *E* compartments were further divided into *S_q_*, *E_cq_* and *E_hq_* by considering specific tracing and quarantine strategies. With the normalization of a series of NPIs such as universal mask use and temperature screening in public places, analyses of the responses to an epidemic rebound may provide important implications for the improvement of control and prevention policies at the local level. However, the study has several limitations. First, information was missing for a small proportion of infected individuals, including demographic characteristics and dates of exposure, which might have led to biased estimates of incubation period. Second, we did not consider heterogeneity among the populations. Future modelling studies may further take age or race into account. Third, due to our focus on differential NPIs, we simplified the assumption with regard to *R* compartment that is associated with medical and nursing resources. Our estimate of recovery rate was similar to a previous study [34], indicating that our model well accommodated to the real-world transmission patterns.

## Conclusions

We generated an SEIAR transmission model by taking into account asymptomatic infection and staged NPIs, such as tracing and quarantine strategies. When a COVID-19 cluster occurs, comprehensive quarantine measures are more effective in preventing community transmission than other individual interventions. Regions with tense resources can adopt a strict home quarantine for exposed individuals as it is a relatively affordable and low-impact intervention compared to centralized quarantine. Management strategies should be continuously adjusted in response to the changing trends of the pandemic accounting for local epidemiology, economy, vaccination level and the strength of health systems.

## Supporting information

Jiang et al. supplementary material Jiang et al. supplementary material

## Data Availability

Data supporting the findings of this study are publicly available from the official websites of China, including the Health Commission of Shaoxing (http://sxws.sx.gov.cn/) and WeChat public account of Shaoxing government (http://m.shaoxing.com.cn/z/2881390/).

## Abbreviations

COVID-19: coronavirus disease 2019
L-BFGS: limited-memory Broyden-Fletcher-Goldfarb-Shanno
LooIC: Leave-One-Out Information Criterion
NAAT: nucleic acid amplification test
NPI: non-pharmacological intervention
SARS-CoV-2: severe acute respiratory syndrome coronavirus 2
SEIAR: susceptible-exposed-infectious-asymptomatic-recovered
SEIR: susceptible-exposed-infectious-recovered

## Supplementary material

For supplementary material accompanying this paper visit http://doi.org/10.1017/S0950268823001796. click here to view supplementary material
