# Supplementary material for: Evaluation of phase-adjusted interventions for COVID-19 using an improved SEIR model
Source: Epidemiol Infect. 2023 Nov 13;152:e9. doi: 10.1017/S0950268823001796 (PMC10789923; doi:10.1017/S0950268823001796)
Supplement: Jiang et al. supplementary material [file S0950268823001796sup001.docx]

**Supplementary Materials**

**Interventions in Shaoxing**

Shaoxing reported the first case with positive COVID-19 infection on December 7, 2021. Since then, serial non-pharmacological interventions have been implemented to control this outbreak. The outbreak basically ended in late December with no newly infected cases being detected. For further analyses of the impacts of these interventions, we divided the implementation of interventions from December 7 to 31 into three stages based on the dates of key events. The first stage was from December 7 to 10, 2021, the second stage was from December 11 to 15, 2021, and the third stage was from December 16 to 31, 2021. Details of main measures for COVID-19 containment are listed in Fig. S1 and Table S1.


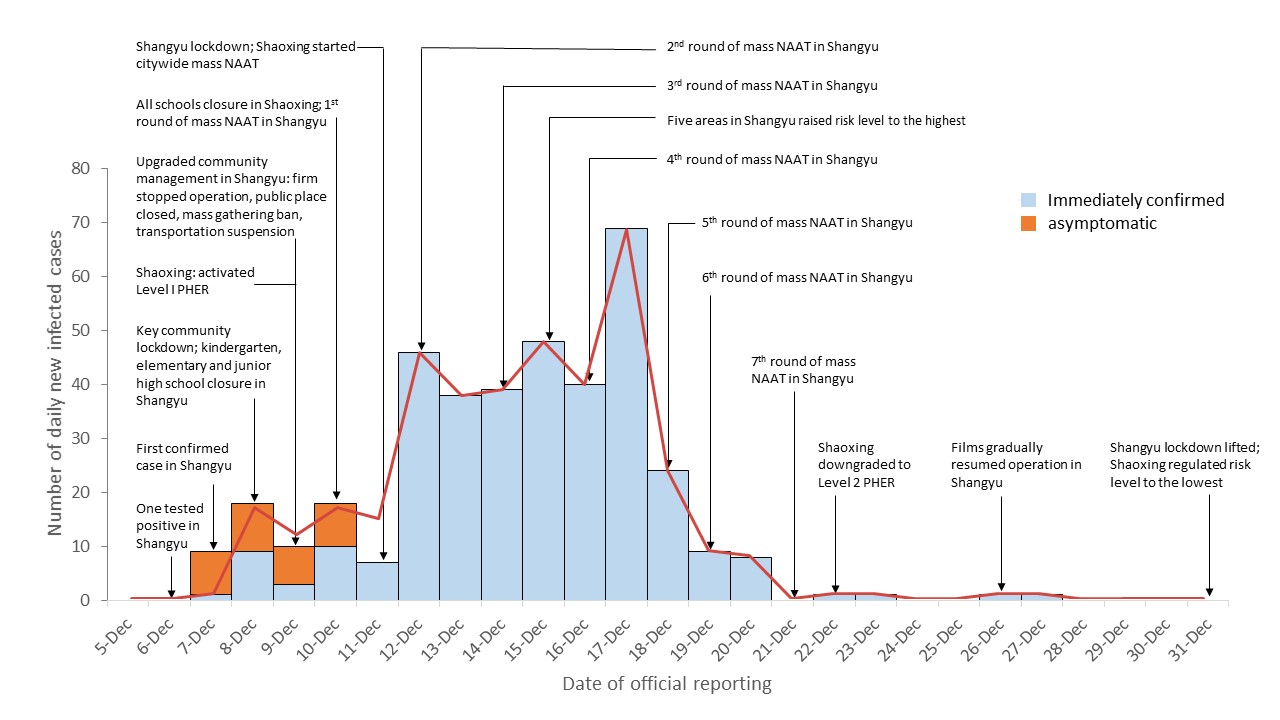


**Fig. S1. The daily new COVID-19 infections and the implementation of interventions in Shaoxing epidemic.** We present the data of infections from December 5 to 31, 2021, with the details of main interventions implemented during this period. The blue column represents the number of daily new immediately confirmed cases; the orange column represents the number of daily new asymptomatic carriers (all of them developed symptoms subsequently, and thus defined as later confirmed cases in this study); the red line shows the trend of the total number of daily new infections.

**Table S1. The implementation of main interventions for COVID-19 in Shaoxing.**

| **Start date** | **Control measures** |
| --- | --- |
| 2021/12/7 | Mass NAAT was conducted in sealed-off, control and prevention areas. |
| 2021/12/8 | Shangyu District upgraded COVID-19 emergency response to the highest level (Level One PHER), with strict measures including mass gathering ban, suspension of kindergartens, primary schools and high schools, and close management on welfare, pension and mental health institutions. |
| 2021/12/9 | Shangyu District strengthened community management by imposing strict home isolation in sealed-off areas, transportation suspension, and closure of business units and public places. |
|  | Shaoxing and Yuecheng District activated Level One PHER for COVID-19. |
| 2021/12/10 | Closed-off management was imposed on schools and off-campus training institutions of all levels in Shaoxing. |
|  | First round of mass NAAT was conducted in Shangyu District from December 10 to 11, 2021. |
|  | Emergency medical teams for nucleic acid sampling dispatched from other cities in Zhejiang to Shangyu District. |
| 2021/12/11 | Shangyu District was locked down and all entrances and exits were closed. |
|  | A city-wide NAAT was activated in Shaoxing. |
| 2021/12/12 | First round of mass NAAT was conducted in Yuecheng District from December 12 to 15, 2021. |
|  | Second round of mass NAAT was conducted in Shangyu District from December 12 to 13, 2021. |
| 2021/12/13 | A negative NAAT certificate within 48 hours was required for entering (returning) Shaoxing or leaving Zhejiang Province. |
|  | Three Fangcang laboratories for testing were built and put into use. |
| 2021/12/14 | Third round of mass NAAT was conducted in Shangyu District from December 14 to 15, 2021. |
| 2021/12/15 | Risk level for COVID-19 in five areas in Shangyu District was raised to the highest level. |
| 2021/12/16 | Fourth round of mass NAAT was conducted in Shangyu District from December 16 to 17, 2021. |
|  | The first batch of 5025 sets of centralized isolation houses in Shaoxing was built. |
| 2021/12/17 | Second round of mass NAAT was conducted in Yuecheng District from December 17 to 18, 2021. |
|  | The largest temporary quarantine site with 600 beds in Shangyu District was built and delivered. |
| 2021/12/18 | Fifth round of mass NAAT was conducted in Shangyu District. |
| 2021/12/19 | Sixth round of mass NAAT was conducted in Shangyu District. |
| 2021/12/21 | Seventh round of mass NAAT was conducted in Shangyu District. |
| 2021/12/22 | Shaoxing and Yuecheng District downgraded PHER to Level Two. |
| 2021/12/24 | Schools in Yuecheng District reopened face-to-face classes. |
| 2021/12/26 | Firms gradually resumed operation in Shangyu District. |
| 2021/12/27 | The lockdown in Yuecheng District was lifted. |
| 2021/12/31 | Shangyu District lifted lockdown and downgraded PHER to Level Two. |
|  | Shaoxing downgraded PHER to Level Three and regulated its risk level to the lowest. |
| 2022/1/2 | Kindergartens, primary schools and high schools in Shangyu District orderly reopened face-to-face classes from Jan 2, 2022 to Jan 10, 2022. |

Abbreviation: NAAT, nucleic acid amplification test; PHER, public health emergency response.

**Estimation of incubation period**

The incubation period (days) was estimated for all infected cases, immediately confirmed cases, and later confirmed cases, respectively. Three distributions (Weibull, Gamma, and Lognormal distribution) were fitted using Hamiltonian Monte Carlo method for Bayesian Inference.


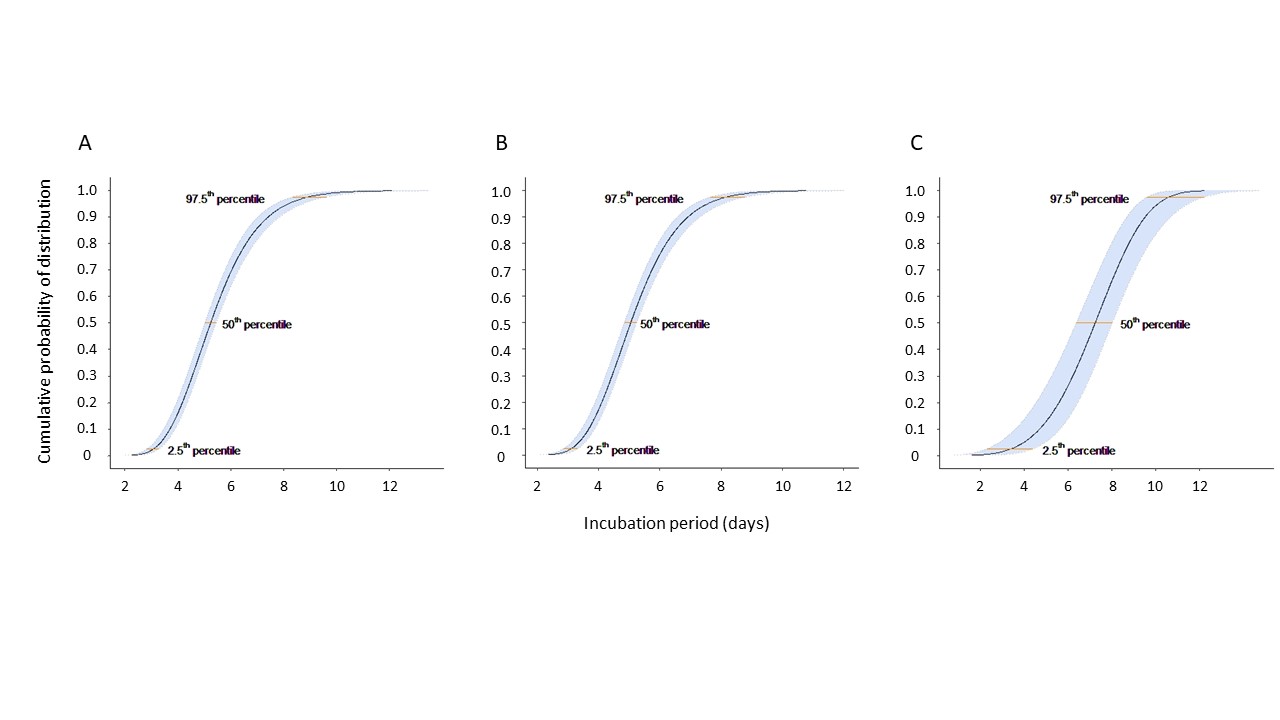


**Fig. S2. Cumulative probability distribution of the estimated incubation period.** Three parametric distributions (Weibull, Gamma, and Lognormal distribution) were used to fit for the incubation period for all infected cases, immediately confirmed cases, and later confirmed cases. We present the best fitted parametric distribution for each group of COVID-19 cases. Here, A) all infected cases (Lognormal distribution); B) immediately confirmed cases (Lognormal distribution); C) later confirmed cases (Weibull distribution).

**Table S2. Percentiles of the estimated incubation period for COVID-19 cases.**

| **Percentiles** | **Weibull** | | **Gamma** | | **Lognormal** | |
| --- | --- | --- | --- | --- | --- | --- |
|  | **Mean (d)** | **95% CI** | **Mean (d)** | **95% CI** | **Mean (d)** | **95% CI** |
| **All infected cases (n=204)** | | | | | | |
| 2.5^th^ | 2.2 | 2.0-2.5 | 3.0 | 2.7-3.2 | 3.1 | 2.8-3.3 |
| 5^th^ | 2.7 | 2.4-3.0 | 3.3 | 3.0-3.5 | 3.4 | 3.1-3.6 |
| 50^th^ | 5.4 | 5.2-5.7 | 5.3 | 5.1-5.5 | 5.2 | 5.0-5.5 |
| 95^th^ | 8.0 | 7.7-8.5 | 8.0 | 7.6-8.5 | 8.2 | 7.7-8.7 |
| 97.5^th^ | 8.5 | 8.1-9.0 | 8.6 | 8.2-9.2 | 8.9 | 8.3-9.6 |
| 99^th^ | 9.0 | 8.6-9.6 | 9.4 | 8.8-10.0 | 9.8 | 9.1-10.7 |
| **Immediately confirmed cases (n=175)** | | | | | | |
| 2.5^th^ | 2.4 | 2.1-2.7 | 3.0 | 2.8-3.3 | 3.1 | 2.9-3.3 |
| 5^th^ | 2.8 | 2.5-3.1 | 3.3 | 3.1-3.6 | 3.4 | 3.1-3.6 |
| 50^th^ | 5.2 | 5.0-5.5 | 5.1 | 4.9-5.3 | 5.0 | 4.8-5.3 |
| 95^th^ | 7.4 | 7.1-7.8 | 7.4 | 7.1-7.9 | 7.6 | 7.2-8.1 |
| 97.5^th^ | 7.8 | 7.5-8.2 | 7.9 | 7.5-8.5 | 8.2 | 7.7-8.8 |
| 99^th^ | 8.2 | 7.9-8.7 | 8.6 | 8.1-9.2 | 8.9 | 8.3-9.7 |
| **Later confirmed cases (n=29)** | | | | | | |
| 2.5^th^ | 3.4 | 2.3-4.4 | 3.9 | 3.0-4.7 | 3.7 | 2.8-4.5 |
| 5^th^ | 4.0 | 2.9-5.0 | 4.3 | 3.4-5.1 | 4.1 | 3.2-4.8 |
| 50^th^ | 7.2 | 6.4-8.0 | 6.9 | 6.1-7.7 | 6.8 | 5.9-7.7 |
| 95^th^ | 10.1 | 9.2-11.5 | 10.3 | 9.1-12.1 | 11.2 | 9.5-14.0 |
| 97.5^th^ | 10.6 | 9.6-12.2 | 11.1 | 9.7-12.2 | 12.3 | 10.3-15.9 |
| 99^th^ | 11.2 | 10.1-13.1 | 12.0 | 10.4-14.6 | 13.8 | 11.3-18.5 |

We present the mean values and 95% confidence interval (CI) of incubation period for each percentile of estimates.

Abbreviation: CI, confidence interval.

**Model fitting and scenario simulation**

We established an extended SEIR (susceptible-exposed-infectious-recovered) model to SEIAR (susceptible-exposed-infectious-asymptomatic-recovered), with four novel compartments (*A, E_cq_, E_hq_, S_hq_*) introduced. The total number of populations in Shaoxing was obtained from local official website. We assumed the whole population were initially susceptible. The number of populations in other compartments were assumed according to the epidemic situation. Transition rate of latent infection to positive infection (*α*) was calculated based on our estimates of incubation period (1/*α*). The isolation period (1/*λ*) was set as 21 days according to local quarantine policy. Two parameters, *q* and *p*, were calculated based on the actual effects of intervention implementation. Other parameters were estimated using the Limited-Memory Broyden-Fletcher-Goldfarb-Shanno (L-BFGS) algorithm.

**Table S3. Effects of interventions, estimated daily infected individuals and the reproduction number during three stages of intervention.**

| **Stage** | **Period** | ***q*** | ***p*** | **Estimated *β* (95% CI)** | ***R*** |
| --- | --- | --- | --- | --- | --- |
| First stage | 2021/12/7 – 2021/12/10 | 0.8085 | 0.3333 | 2.22356 (2.22354-2.22357) | 26.2183 |
| Second stage | 2021/12/11 – 2021/12/15 | 0.3978 | 0.8929 | 3.93563 (3.93562-3. 93563) | 46.9048 |
| Third stage | 2021/12/16 – 2021/12/31 | 0.9870 | 1.0000 | 1.98448 (1.98448-1.98449) | 23.2556 |

*q* and *p* are two main parameters that inform the effectiveness of interventions. Specifically, *q* represents the probability of an exposed person being traced and centralized quarantined; *p* represents the probability of an exposed people being home-quarantined; *β* reflects the transmission velocity that was estimated by model fitting; *R* is the basic reproduction number.

Abbreviation: CI, confidence interval.

**Table S4. The definitions and initial values of model parameters.**

|  | **Definition** | **Value** | **Source** |
| --- | --- | --- | --- |
| N | Total population of Shaoxing | 5.27 million | Ref [1] |
| S | Susceptible people | 5.27 million | Ref [2] |
| S_q_ | Comprehensive quarantined susceptible people | 0 | Assumed |
| E | Exposed people | 9 | Assumed |
| E_hq_ | Exposed people under home quarantine | 0 | Assumed |
| E_cq_ | Exposed people under centralized quarantine | 0 | Assumed |
| I | Symptomatic infected people | 1 | Assumed |
| A | Initially asymptomatic infected people | 0 | Assumed |
| R | Removed from model (recovered or deceased) | 0 | Assumed |
| Q | The number of people quarantined per day | 606310 | Estimated |
| ε | The transmission coefficient of exposed people compared with symptomatic people | 0.5728 | Estimated |
| θ | The transmission coefficient of asymptomatic people compared with symptomatic people | 0.5841 | Estimated |
| α | The transition rate of latent infection to positive infection | 1/5.4 | Computed |
| λ | The rate of release from quarantine/isolation | 1/21 | Ref [2] |
| η | The proportion of symptomatic people in positive infections | 355/387 | Computed |
| γ | The rate of recovery or death | 0.0824 | Estimated |
| q | The probability of an exposed person being traced and under centralized quarantine | 38/47 | Computed |
| p | The probability of an exposed people being home-quarantined | 3/9 | Computed |
| β(t) | The number of infected persons over time | 2.22356 | Estimated |

We present the meanings, initial values, and the sources of each compartment and parameter in the transmission model. Here, *q*, *p* and *β* are shown with their computed/estimated values in the first stage of intervention.

**Supplementary References**

1. Shaoxing Municipal Bureau of Statistics. Data bulletin of the seventh census of Shaoxing. Feb 3, 2022. <http://tjj.sx.gov.cn/art/2021/5/18/art_1229362069_3865654.html>.

2. Shaoxing Health Commission. The latest update on the epidemic situation in Shaoxing. Jan 30, 2022.
